# Correlation between Selective Motor Control of the Lower Extremities and Balance in Spastic Hemiplegic Cerebral Palsy: a randomized controlled trial

**DOI:** 10.1186/s13102-023-00636-0

**Published:** 2023-03-05

**Authors:** Amira H. Mohammed, Hager R. El-Serougy, Amel E Abdel Karim, Mohamad Sakr, Samah M. Sheha

**Affiliations:** 1 Department of Physical Therapy for Pediatric and its Surgery, Faculty of Physical Therapy, Delta University for Science and Technology, Gamasa, Egypt; 2 Department of Physical Therapy for Neuromuscular Diseases and its Surgery, College of Physical Therapy, Misr University for Science and Technology, Giza, Egypt; 3 Department of Physical Therapy for Pediatric Diseases and its Surgery, College of Physical Therapy, Misr University for Science and Technology, 77, Giza, Egypt; 4 Department of Neurology, College of Medicine, Misr University for Science and Technology, 77, Giza, Egypt

**Keywords:** Balance, Children, Mirror therapy, Selective motor control, Spasticity

## Abstract

**Background:**

Children with cerebral palsy (CP) have motor deficits caused by spasticity, weakness, contractures, diminished selective motor control (SMC), and poor balance. The purpose of the current study was to evaluate the influence of mirror feedback on lower extremity selective motor control and balance in children with hemiplegic cerebral palsy. Understanding the relationship between SMC and balance will help children with hemiplegic CP receive more appropriate therapies.

**Methods:**

Forty-seven children of both sexes diagnosed with hemiplegic CP participated in the study. Group1 (Gr1 - control group) received conventional physical therapy training while group 2 (Gr2 - intervention group) received conventional physical therapy training in addition to bilateral lower extremity mirror therapy (MT). The primary outcome measure used was Selective Control Assessment of Lower Extremity scale (SCALE), while the secondary outcome measure was the Pediatric Balance Scale (PBS).

**Results:**

There were significant differences in Selective Control Assessment of Lower Extremity Scale (SCALE) and Pediatric Balance Scale (PBS) between both groups in favor of Gr2. After treatment, both groups improved significantly, yet Gr2 outperformed Gr1 by a large margin.

**Conclusion:**

Mirror therapy may be a useful addition to home-based motor interventions for children with hemiplegic CP due to its relative simplicity, low cost, and high patient adherence. Additionally, it may help children improve their selective motor skills and balance.

**Trial registration:**

Current Controlled Trials using African Clinical Trials Registry website with ID number PACTR202105604636415 retrospectively registered on 21/01/202.

## Background

The most prevalent mobility disorder in children is cerebral palsy (CP), which has an average frequency of three per 1000 live births worldwide and a higher prevalence of 60 to 150 per 1000 among preterm infants who are born weighing less than 1500 g [1].

The three basic categories of CP are ataxic, dyskinetic, and spastic. Approximately 87% of children with CP are in the spastic category [2]. Selective motor control (SMC), which provides quick, autonomous regulation of joint mobility, is a crucial component of typical human movement. In spastic CP, impaired SMC is one of four connected neuromuscular impairments that frequently coexist with muscle weakness, spasticity, and short muscle-tendon length. Injury to the corticospinal tract (CST) and other descending motor tracts, and the ensuing loss of descending excitatory and inhibitory signals, are linked to all four motor impairments [3].

“Impaired ability to actively contract individual muscles in a specified pattern in response to demands of a voluntary posture or movement” is the definition of impaired SMC. A pattern of flexor or extensor movement at two or more joints is referred to as a synergistic mass movement pattern. These flexor or extensor synergies prevent isolated joint movements, forcing flexor or extensor muscles to work together (co-activation) during functional activities like walking [4]. The loss of SMC inhibits motor function more than other impairments such as spasticity and/or contractures, according to studies examining the consequences of different motor impairments in children with CP [5].

Children with cerebral palsy can benefit from a variety of therapeutic approaches, including neurodevelopmental therapies, bilateral therapeutic exercises, constraint-induced movement therapy, sensory integration therapy, and mirror therapy [6].

Modern methods for regaining function of the more affected upper extremity (UE) after a stroke include mirror therapy. Mirror therapy is based on visual stimulation, in contrast to other therapies, which use somatosensory input to aid motor recovery. A mirror is held in the patient’s midsagittal plane during mirror treatment, reflecting the less-affected side as if it were the more affected side. In this configuration, the less affected extremity movements provide the appearance that the more affected extremity is moving normally. The comparatively simple administration and potential for self-administered home therapy, especially for those with severe motor deficiencies, are two benefits of mirror therapy [7, 8]. Visual feedback in hemiparesis improves the congruence between afferent (visual) feedback, boosts the excitability of the ipsilesional motor cortex, and encourages an internal representation of motor motions [9]. Studies show that coordinated movements of the affected and unaffected extremities alter interhemispheric inhibition, enabling the unaffected hemisphere to assist the affected hemisphere in becoming activated [10].

Mirror therapy is simple to use, affordable, and non-invasive. Thus, it can be considered a promising and safe addition to hemiparesis therapy in youngsters [11]. It has been the subject of several experimental trials for the treatment of UE pain and motor rehabilitation after stroke. The use of MT for treatment of lower extremities (LE) has received little consideration [12].

Therefore, the purpose of this study was to evaluate the influence of mirror feedback on lower extremity selective motor control and balance in children with hemiplegic cerebral palsy. Understanding the relationship between SMC and balance will support in the delivery of more appropriate therapies to children with hemiplegic CP. Combining easier and more accessible alternative therapies with traditional rehabilitation programs may provide additional benefits for improving LE motor function.

## Methods

### Study design

A randomized controlled trial was carried out at the outpatient clinics for pediatric rehabilitation – College of Physical Therapy, Cairo University between the period of December 2018 to October 2021. The study was registered retrospectively on the Pan African Clinical Trials Registry with number of PACTR202105604636415 date 21/01/2021.

Procedures of the current study agreed with the ethical code stated in Helsinki Declaration 1975 and was approved by the Ethical Committee Board of the College of Physical Therapy - Cairo University (number: P.T.REC/012/003118). All procedures for assessment and interventions were carried out at the outpatient clinics for pediatric rehabilitation – College of Physical Therapy, Cairo University.

All parents were given a summary of the evaluation and treatment methods, after which they signed an informed consent form stating “the information in this study has been read and explained to me besides reading it myself. I’ve had the chance to ask questions regarding it, and every one of them has received satisfactory answers. I freely give my child my consent to take part in this study”.

## Participants

Sixty-five **(n = 65)** children with hemiplegic CP were recruited, eight **(n = 8)** were excluded for not meeting the criteria, three parents **(n = 3)** refused to provide their consent and the remaining fifty-four **(n = 54)** were randomly assigned to two equal groups (Gr1 - control group and Gr2 - intervention group). During rehabilitation, seven children **(n = 7)** discontinued the intervention; five children **(n = 5)** travelled with their parents to another city and two children **(n = 2)** did not participate in the post-treatment evaluation. Inclusion criteria were as follows: (1) diagnosis of hemiplegic CP (2) age range six to nine years, (3) mild to moderate spasticity of the more affected side (score 1–2) measured by the Modified Ashworth Scale (MAS) (4) adequate cognitive and linguistic abilities (e.g., working memory, attention, and concentration), required for focusing on mirror reflection, for at least ten minutes and follow the therapist’s directions and (5) normal and pain free range of motion (ROM) of the less affected LE. Children who had fixed deformities in the LE that interfered with motor functions, history of epilepsy and/or surgical interference or Botulin toxin injection in the LE within the previous year and un-cooperative were excluded from the study.

## Randomization

The sample size was determined using data from prior research published in 2017. Researchers assessed the effect of MT on gross motor skills in children with spastic CP. Based on findings, there were substantial changes between the mirror treatment group and the control group. Using their data and G*Power (version 3.1.9.2; Germany) were used to compute the sample size [13]. A total of 54 were recruited to compensate for possible dropouts from the study (13% drop rate n = 7).

**Fig. 1:**
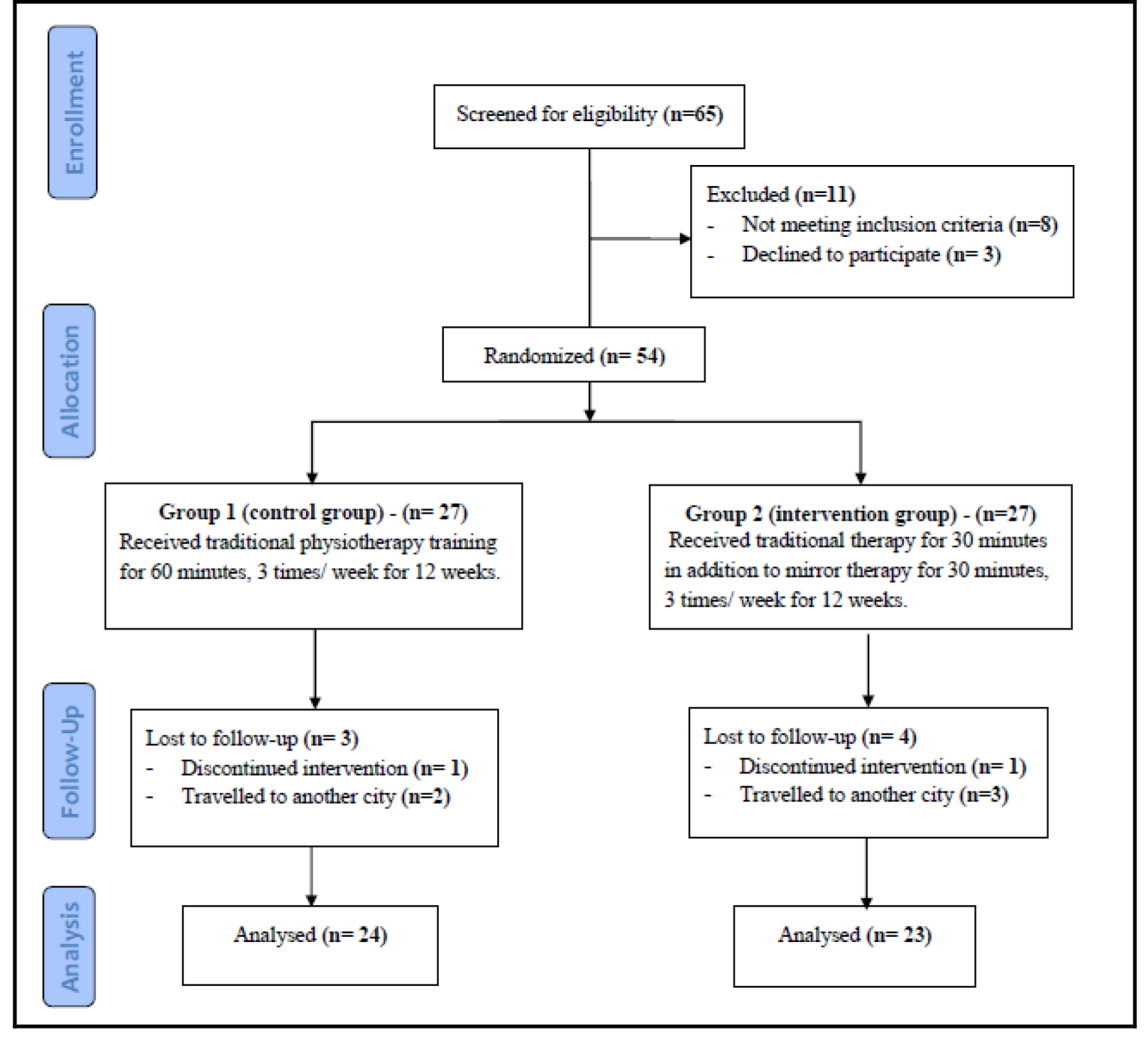
Flow Diagram of the Study

All hemiplegic CP children were randomly assigned to two equal groups, using a random allocation software. Children were divided to two equal groups using the Graph Pad Quick Calcs website [14], (n = 27 in each group); Group1 (Gr1 - control group) received conventional physical therapy training while group 2 (Gr2 - intervention group) received conventional physical therapy training in addition to bilateral lower extremity MT. Figure 1 shows a representation of the children’s retention and randomization across the study. In the present investigation, a single-blind approach was adopted, in which only study participants were made aware of their participation.

## Outcome measurements

***Primary outcome measure***: Selective Control Assessment of Lower Extremity Scale (SCALE) was used to evaluate the ability of patients with spasticity to perform SMC movements in different joints. It was used to evaluate toe, subtalar, ankle, knee, and hip joints range of motions (ROM). Except for hip joint flexion, which was examined in the side-lying position to obtain appropriate joint excursion, other measurements were done in the sitting position. The children were taught to perform the task by counting for three seconds while the examiner passively moved their extremities through the required ROM. After the child completed the required movement in time without moving any untested ipsilateral or contralateral LE joints, a score of 2 points “normal” was given. A child earned score 1 “impaired” if he or she performed one of the following errors: just one directional movement, less than 50% of movement achieved, movement of a non-tested joint (including mirror motions), or time beyond a three-second spoken count. The child earned a 0 score “unable” when he/she did not begin the necessary movement or perform a mass extensor or flexor synergistic pattern [15]. This was repeated for each joint in the LE.

### Secondary outcome measure

The Pediatric Balance Scale (PBS) is a widely used instrument for examining children with CP who have a difficulty maintaining standing balance. It is made up of 14 three-dimensional components that include standing, sitting, and postural adjustments. Each item is graded on a scale of 0 to 4, with a higher score indicating greater balance [16]. The PBS and SCALE were used twice for each child, at baseline and three months following intervention.

## Therapeutic intervention

Children in Gr1 (control group) received conventional therapy for 60 min while children in group 2 (intervention group) received MT and conventional physiotherapy for 60 min (30 min MT and 30 min conventional therapy). Both groups received three sessions per week for 12 consecutive weeks. The conventional therapy included stride standing allowing weight shift from one extremity to another, standing on one foot, stoop, and recovery from standing position, standing on balance board, balance training exercises, stretching exercises for hip joint flexors, adductors, hamstrings, and calf muscles and gait training exercises in an open environment [17, 18].

To implement MT, birthmarks and scars on the less affected LE were covered prior to treatment if they impaired the ability to see clearly. During administration of MT, the atmosphere was free of any distractions, to ensure that each child focused and paid attention. A mirror was placed between both LE, with the more affected LE hidden behind a black curtain and the reflection of the less affected LE completely visible. The mirror’s dimensions were large enough at least 35 × 25 inches [19] to cover the entire more affected LE and allow children to see all movements in the mirror.

The child was then told to watch the mirror reflection for a minute or two while attempting to imagine the mirror image of the more affected LE. Instructions were given to perform the movement, after the therapist first visually demonstrated it with the less affected LE [19].

In sitting position, each child was asked to perform simultaneous bilateral toe flexion/extension, subtalar joint inversion / eversion, ankle joint dorsiflexion /plantarflexion, knee joint flexion/extension, and in long sitting simultaneous bilateral hip joint adduction/abduction and flexion/extension. If symptoms such as lightheadedness, nausea, or perspiration apperaed, the child was told to focus on the less affected LE or another area of the room instead of the mirror. The child was then told to look at the mirror image for a brief length of time before shifting sight to the less affected LE. This process was repeated several times till any side-effects disappered. The intensity of the mirror illusion was facilitated by moving everything extremely slowly. At the end the treatment session, children were able to move the more affected LE. [19].

### Statistical analysis

Descriptive statistics was used to identify each variable`s mean and standard deviation. Paired t-test was used to compare characteristics of patients between both groups. Chi-square test was used for comparison between two groups in age and gender. The significance level for all the statistical tests was set at p-value ≤ 0.05. IBM statistical software version 21 (Chicago, IL, USA) was used to perform all the statistical analyses.

## Results

### Children characteristics

Table 1 shows the characteristics of children, including age, gender, and degree of spasticity. Children characteristics between both groups did not differ significantly (p > 0.05).

**Table 1:** Comparison mean values of demographic data between both groups

Variables	Gr 1 (n = 24)	Gr 2 (n = 23)	P-value
Age (Year)	6.67 ± 0.70	6.80 ± 0.74	0.630
Gender (M/F)	8/16	8/15	0.856
Degree of spasticity (1/1+/2)	5/12/7	4/11/8	0.905

Numerical Data are expressed as mean ± SD or number (%) P-value > 0.05: non-significant

### Effect of Treatment on Lower Extremity (LE) Selective Motor Control (SMC) and Balance

#### Within group comparison

There was a statistically significant improvement in the hip, knee, ankle, and subtalar joints, total extremity, and PBS score in **Gr2** after treatment compared with those before treatment **(p < 0.05)**. Also, effect size was significant in hip and knee joints, total extremity, and PBS score while effect size was medium in the subtalar joint and Toes. On the other hand, there were no significant changes in the toes the ankle joint in **Gr1 (p > 0.05) (**Table 2**)**.

#### Among group comparison

**Fig. 2:**
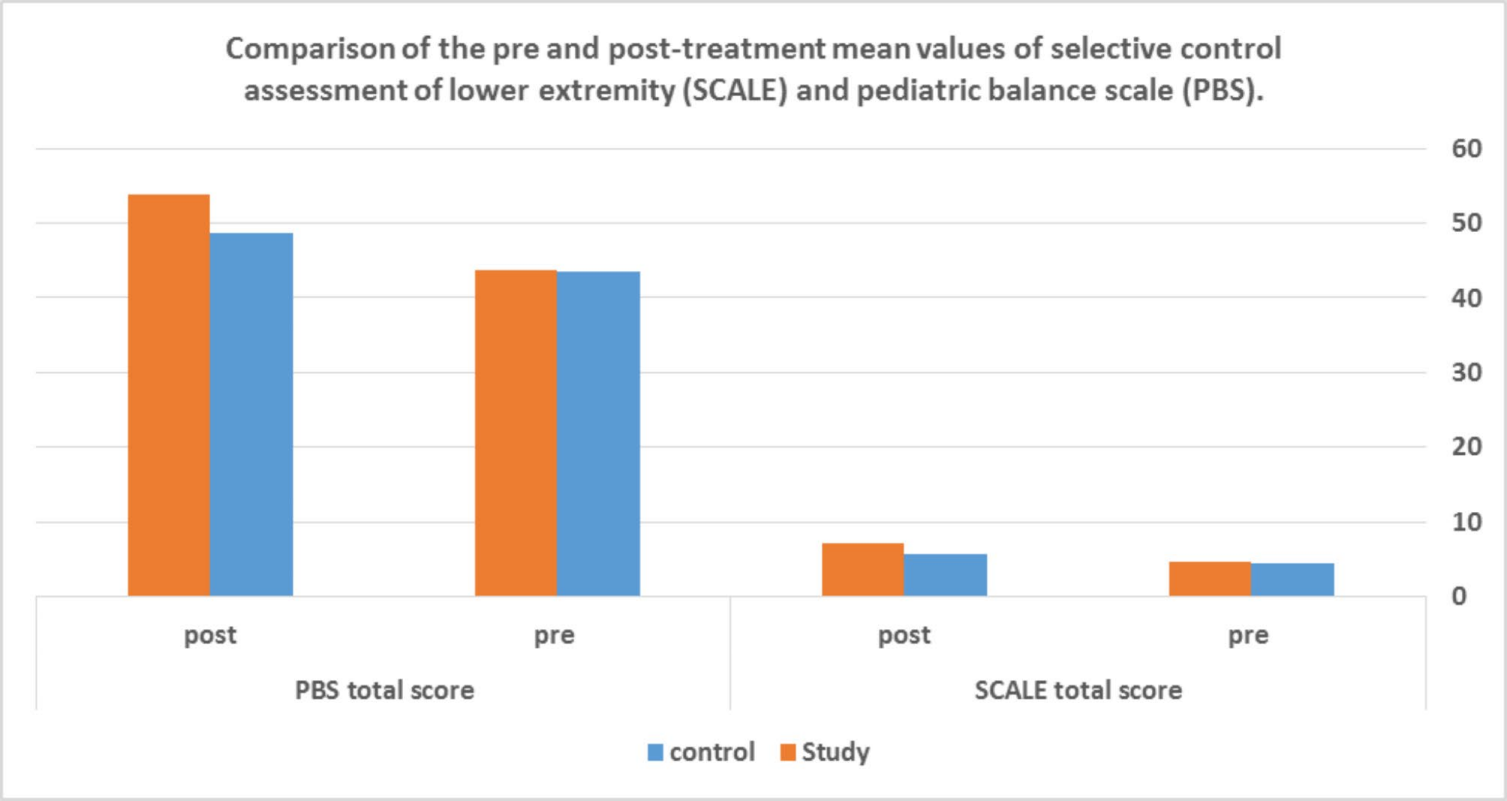
Comparison of pre- and post-treatment values of Selective Control Assessment of Lower Extremity (SCALE) and Pediatric Balance Scale (PBS) in (Gr1) and (Gr2)

There was no statistically significant differences pre-treatment between groups **(p > 0.05)**. There was a significant improvement in the hip, knee, ankle, and subtalar joints, total extremity, and PBS score in **Gr2** after treatment compared to **Gr1** after treatment **(p < 0.05)**. On the other hand, there was no significant difference in the toes of both groups post-treatment **(p > 0.05) (**Table 3**)** and **(**Fig. 2**)**.

### Correlation between Balance and the Selective Motor Control (SMC) of the Lower Extremity (LE)

There was a substantial significant positive association at 5% significance level between the PBS score and the SCALE total score **(**Table 4**)**.

**Table 2:** The design T- test for all dependent measuring variables within and between (Gr1) and (Gr2)

Variables		Groups (Mean ± SD)		T-value	*P*-value
		Gr1 (n = 24)	Gr2 (n = 23)		
**Hip joint**	**Pre-treatment**	1.07 ± 0.25	1.13 ± 0.34	1.00	**0.334**
	**Post-treatment**	1.47 ± 0.50	1.93 ± 0.25	3.50	**0.004**
	**Improvement %**	37.38%	70.80%		
	**Mean Difference**	0.40	0.80		
	**Effect Size (Cohen’s d)**	0.79	1.93		
	**T-value**	3.06	7.48		
	***P*** **-value**	0.009	0.0001*		
**Knee joint**	**Pre-treatment**	1.07 ± 0.25	1.07 ± 0.25	0.00	**1.000**
	**Post-treatment**	1.33 ± 0.47	1.87 ± 0.34	3.22	**0.006**
	**Improvement %**	24.30%	68.22%		
	**Mean Difference**	0.26	0.73		
	**Effect Size (Cohen’s d)**	0.58	1.93		
	**T-value**	2.26	7.48		
	***P*** **-value**	0.041	0.0001*		
**Ankle joint**	**Pre-treatment**	0.87 ± 0.34	0.93 ± 0.25	1.00	**0.334**
	**Post-treatment**	1.07 ± 0.25	1.27 ± 0.44	1.87	**0.042**
	**Improvement %**	22.99%	36.56%		
	**Mean Difference**	0.20	0.34		
	**Effect Size (Cohen’s d)**	0.44	0.68		
	**T-value**	1.74	2.65		
	***P*** **-value**	0.104	0.019		
**Subtalar joint**	**Pre-treatment**	0.87 ± 0.34	0.87 ± 0.34	0.046	**0.756**
	**Post-treatment**	1.07 ± 0.25	1.13 ± 0.34	1.000	**0.005**
	**Improvement %**	22.99%	29.89%		
	**Mean Difference**	0.20	0.26		
	**Effect Size (Cohen’s d)**	0.48	0.58		
	**T-value**	1.87	2.26		
	***P*** **-value**	0.82	0.041		
**Toes**	**Pre-treatment**	0.67 ± 0.47	0.73 ± 0.44	1.000	0.334
	**Post-treatment**	0.73 ± 0.44	0.93 ± 0.25	1.87	0.082
	**Improvement %**	8.96%	27.40%		
	**Mean Difference**	0.06	0.20		
	**Effect Size (Cohen’s d)**	0.25	0.48		
	**T-value**	1.00	1.87		
	***P*** **-value**	0.334	0.082		
**Total Extremity score**	**Pre-treatment**	4.53 ± 0.62	4.80 ± 0.91	1.16	**0.262**
	**Post-treatment**	5.67 ± 0.60	7.20 ± 0.83	5.27	0.0001*
	**Improvement %**	25.17%	50%		
	**Mean Difference**	1.14	2.40		
	**Effect Size (Cohen’s d)**	1.35	2.02		
	**T-value**	5.26	7.86		
	***P*** **-value**	0.0001*	0.0001*		
**Pediatric Balance Scale (PBS)**	**Pre-treatment**	43.53 ± 0.81	43.67 ± 0.94	1.46	**0.164**
	**Post-treatment**	48.67 ± 0.47	53.87 ± 0.82	0.633	0.0001*
	**Improvement %**	13.03%	23.36%		
	**Mean Difference**	5.67	10.20		
	**Effect Size (Cohen’s d)**	4.84	8.45		
	**T-value**	18.75	32.73		
	***P*** **-value**	0.0001*	0.0001*		

SD: standard deviation T-value: hypothesis test statistic P-value: probability value *non- significant (P-value > 0.05)

**Table 3:** Mean difference values of Selective Control Assessment of Lower Extremity (SCALE) and Pediatric Balance Scale (PBS) in (Gr1) and (Gr2)

Variables		Mean Difference	t-value	p value
**Hip**	**Pre-treatment**	0.067	0.592	**0.559**
	**Post-treatment**	0.47	3.130	0.004*
**Knee**	**Pre-treatment**	0.000	0.000	**1.000**
	**Post-treatment**	0.533	3.434	0.002*
**Ankle**	**Pre-treatment**	0.400	2.806	0.001*
	**Post-treatment**	0.200	1.474	**0.152**
**Subtalar**	**Pre-treatment**	0.000	0.000	**1.000**
	**Post-treatment**	0.067	0.592	**0.559**
**Toes**	**Pre-treatment**	0.067	0.386	**0.702**
	**Post-treatment**	0.200	1.474	**0.152**
**Total limb Score**	Pre-treatment	0.267	0.907	**0.372**
	Post-treatment	1.533	5.602	0.000*
**Pediatric Balance Scale (PBS)**	Pre-treatment	0.133	0.402	**0.691**
	Post-treatment	5.200	25.026	0.000*

SD: standard deviation T-value: hypothesis test statistic P-value: probability value *non- significant (P-value > 0.05)

**Table 4:** Correlation between Selective Control Assessment of Lower Extremity (SCALE) and Pediatric Balance Scale (PBS)

Item		SCALE difference
**PBS difference**	Pearson Correlation	0.521^**^
	Sig. (2-tailed)	0.003

* Correlation is significant at the 0.05 level (2-tailed)

**Correlation is significant at the 0.01 level (1-tailed)

## Discussion

Children with CP have different muscle magnitudes and recruitment patterns compared to healthy ones. These variations might affect voluntary muscle recruitment, resulting in motor disability. On a functional level, SMC is regarded as one of the most notable deficiencies affecting gross motor tasks in children with CP [20, 21].

Children age in the current study ranged between six and nine as the visual and vestibular systems usually develop in tandem with the somatosensory system. Between the ages of four to six, the highest conceivable sensory integration takes place, with the child exhibiting sensory changes like those seen in adults between the ages of 7 and l0 years [18, 22].

This study investigated the effect of mirror feedback on LE SMC and balance in children with hemiplegic CP. Results shown significant improvement of SMC of hip and knee joints within and between groups with favor to the **Gr2**.

For SMC of the ankle and subtalar joints and toes, there was significant difference within Gr2 and no significant difference within Gr1. The results of our study proved that MT resulted in substantial improvements in SMC and balance in the more affected LE when compared to conventional therapy. Additionally, a substantial positive association between improved balance and improved SMC in the more affected LE was reported.

The results of our study supported the findings of **Mohammed et al., (2022)** who stated that MT enhance the SMC of the more affected upper extremity (UE) in spastic hemiplegic CP [10, 23]. Mirror therapy applied to the LE improves the ability of the spastic children to maintain their balance [18].

Additionally, findings of our study agree with those of **Mohan et al., (2013)** and **Mohan et al., (2013)**, who showed that MT intervention can restore balance and gait after a stroke [24]. Significant gains in balance abilities among subacute stroke patients were reported by **Tae-sung et al., (2016)** and **Kim, Myoung-Kwon et al., (2016)** [25, 26].

In this study, the term more affected and less affected LE were used instead of affected.

and non-affected terms commonly used. Each cerebral hemisphere controls function of the.

contralateral side of the body. Yet, some nerve fibers carry information to the same side of the body. Therefore, function can be affected in both sides of the body [27–29]. Thus, recently the term “less affected side” and “more affected side are more accurate [29].

The improvement in SMC for proximal segments of more affected LE may be attributed to anatomical relationship that suggests that distal LE pathways are thought to be more susceptible than proximal LE tracts because of how the LE is organized somatotopically in the sensorimotor cortex [30, 31]. According to cross-sectional research, people with CP have a 20–50% lower capacity to actively contract their quadriceps and plantar flexors, and their muscle activation patterns are different, especially for distal agonists [32, 33].

**Wakeling et al. 2007** found that in children with spastic diplegia, aberrant muscle firing occurred more frequently in the distal than the proximal musculature during locomotion. Additionally, the ankle joint has been measured to have higher muscular weakness than more proximal joints [34, 35]. In participants with spastic CP, **Fowler et al.** assessed the proximal to distal distribution of SMC impairment among LE joints. From the hip to the toes, there was a noticeable decline in SCALE ratings [15].

The disrupted motor command and visual sensory feedback loop are usually repaired by MT [8]. The link between motor output (commands supplied to the more affected extremity) and sensory input are restored by MT, which enhances motor control in the more affected extremity (visual feedback of the affected extremity) [36].

What happens is the more affected extremity becomes misled by the optical illusion created by the non-affected or less affected extremity movement reflected in the mirror, and the patients believe that the more affected extremity is moving, which improves the motor function of the more affected extremity [7]. Widespread activity in the ipsilesional hemisphere, including the attention and cognitive centers, bilateral motor cortices, and some elements of the mirror neuron system, as well as a decrease in intracortical inhibition, are all caused by MT [37].

## Conclusion

Mirror therapy can be considered an effective and simple addition to home-based motor intervention for children with hemiplegic CP due to its relative simplicity, low cost, and high patient adherence. Additionally, it may help spastic children to improve their selective motor skills of the more affected LE and balance.

The present study has the following limitations: (1) authors could not apply this rehabilitation programme to other forms of spastic CP since the effect of MT was studied among spastic hemiplegic CP children with sufficient cognitive ability, (2) the children’s SMC and balance were examined three months after the intervention; as a result, we are unable to make any conclusions regarding the brain’s neural activity at the beginning of therapy and three months afterwards. Additionally, the effects of short-term MT usage were not examined in this study, (3) the effect of the MT on the other functional activities for LE, such as gait, running, and coordination, could not be quantified. So, we recommended further studies to investigate the influence of MT on functional activities for LE, such as gait, running, and coordination.

## Data Availability

The datasets used and/or analyzed during the current study available from the corresponding author on reasonable request.

## Declarations

All parents were given a summary of the evaluation and treatment methods, after which they signed an informed consent form stating “the information in this study has been read and explained to me besides reading it myself. I’ve had the chance to ask questions regarding it, and every one of them has received satisfactory answers. I freely give my child my consent to take part in this study”.

## Abbreviations

CP: cerebral palsy
SMC: selective motor control
MT: mirror therapy
UE: upper extremity
LE: lower extremities
MAS: Modified Ashworth Scale
SCALE: Selective Control Assessment of Lower Extremity Scale, PBS:Pediatric Balance Scale.
